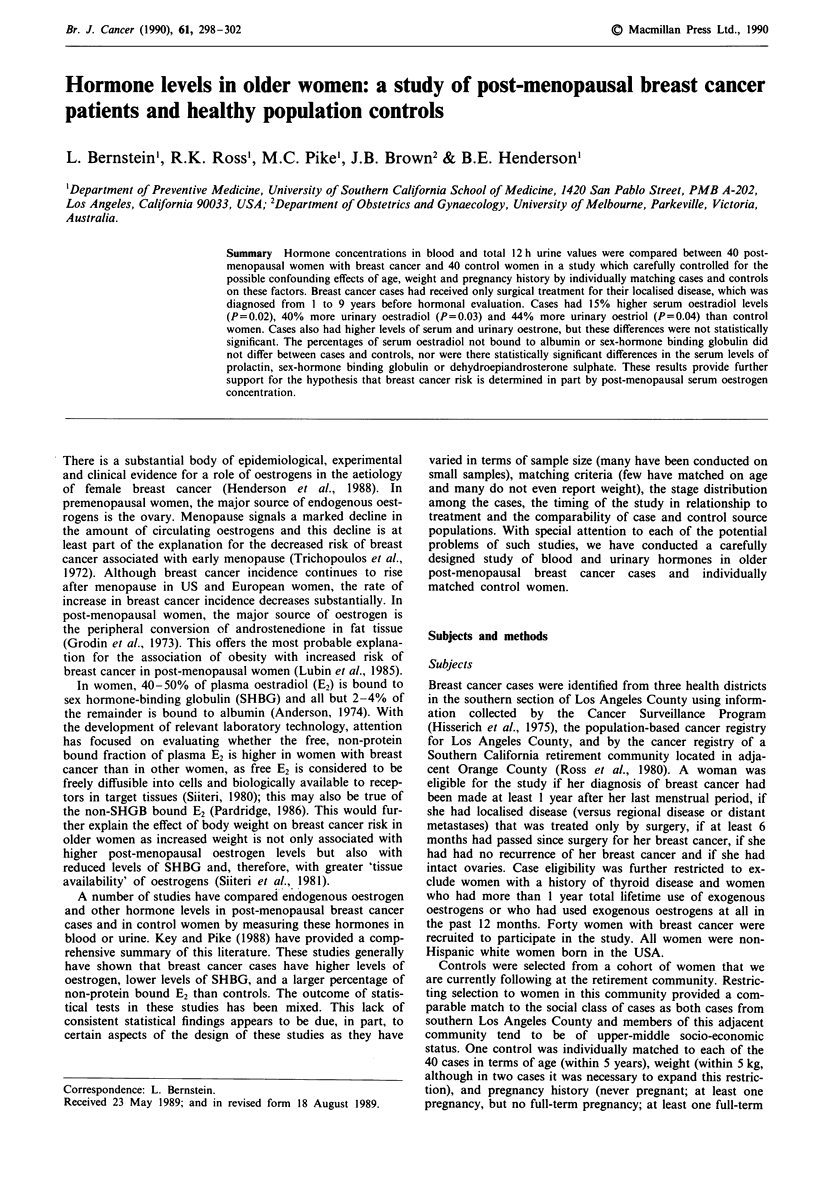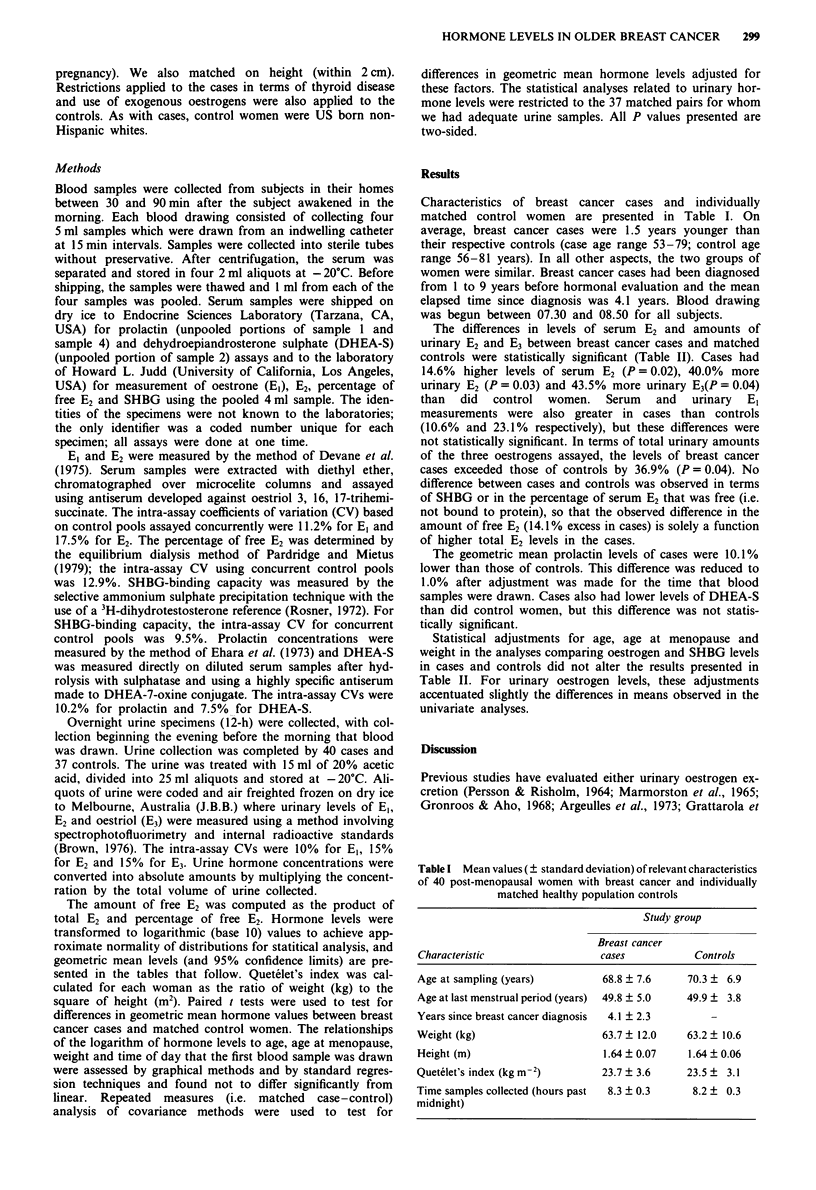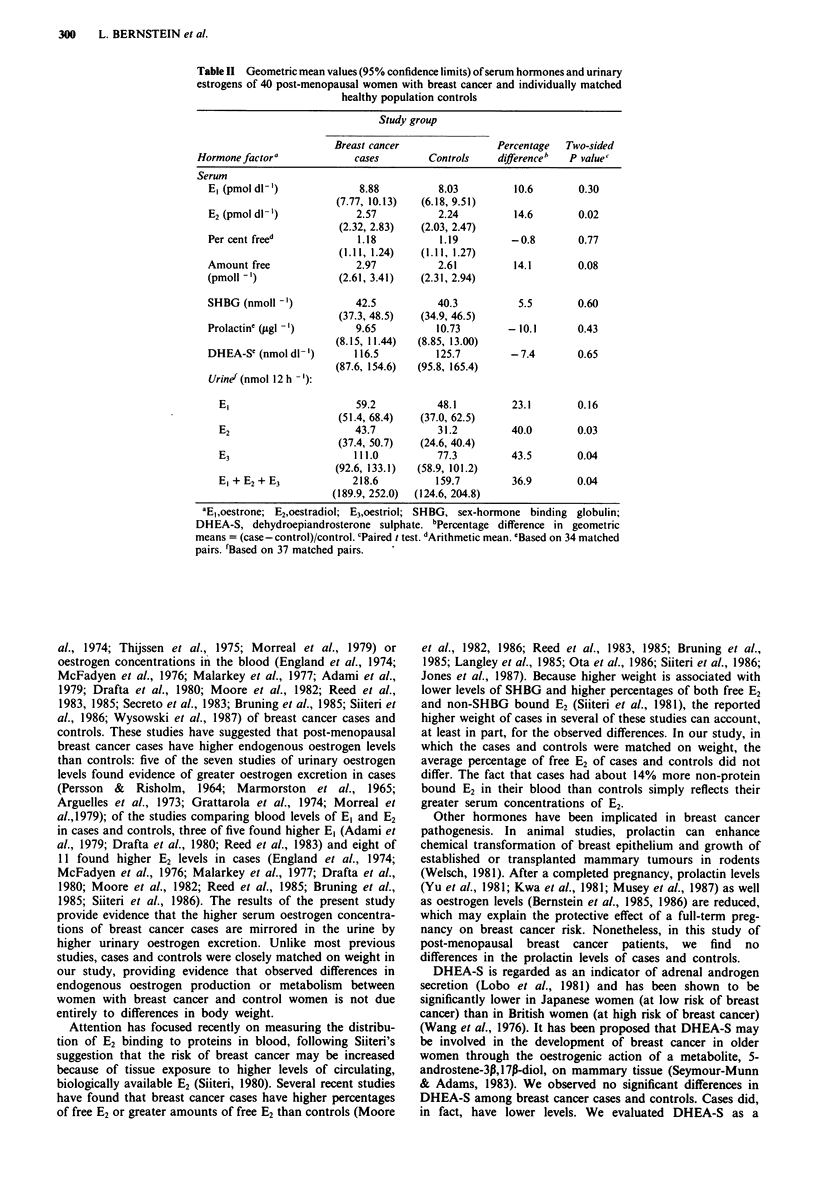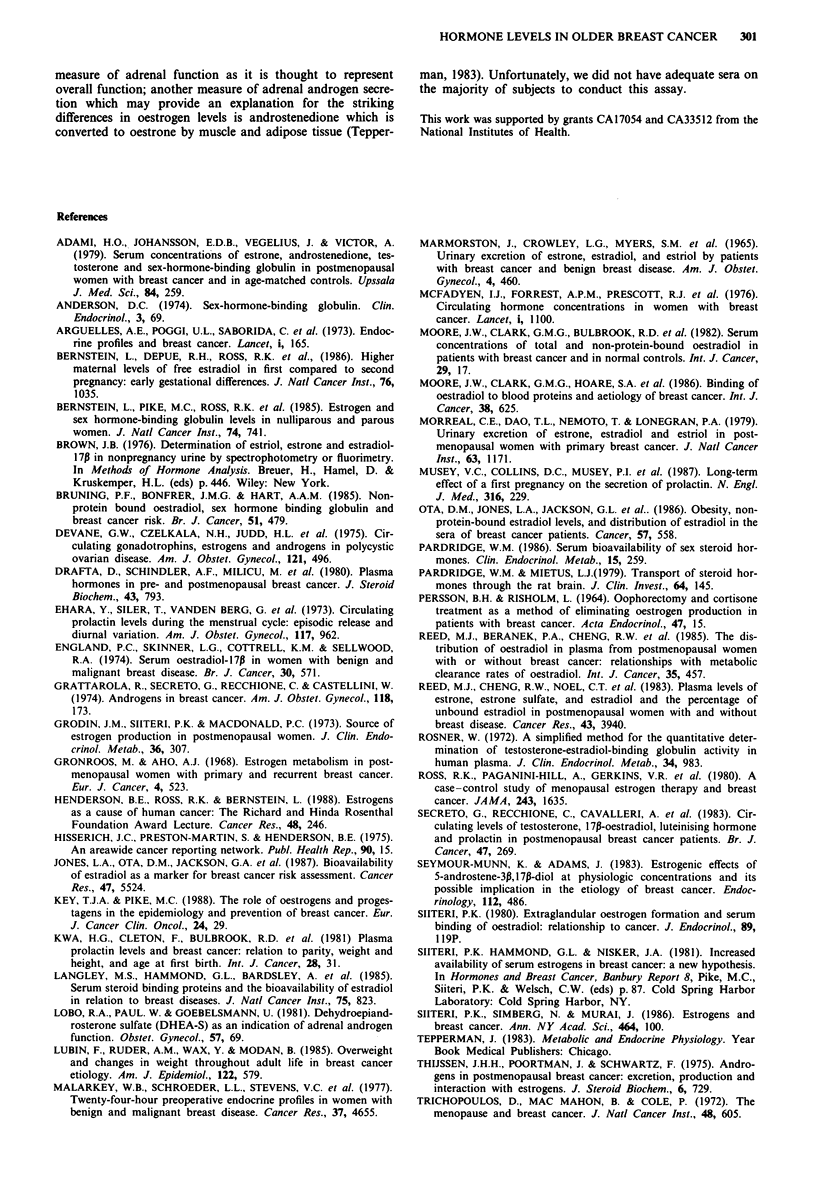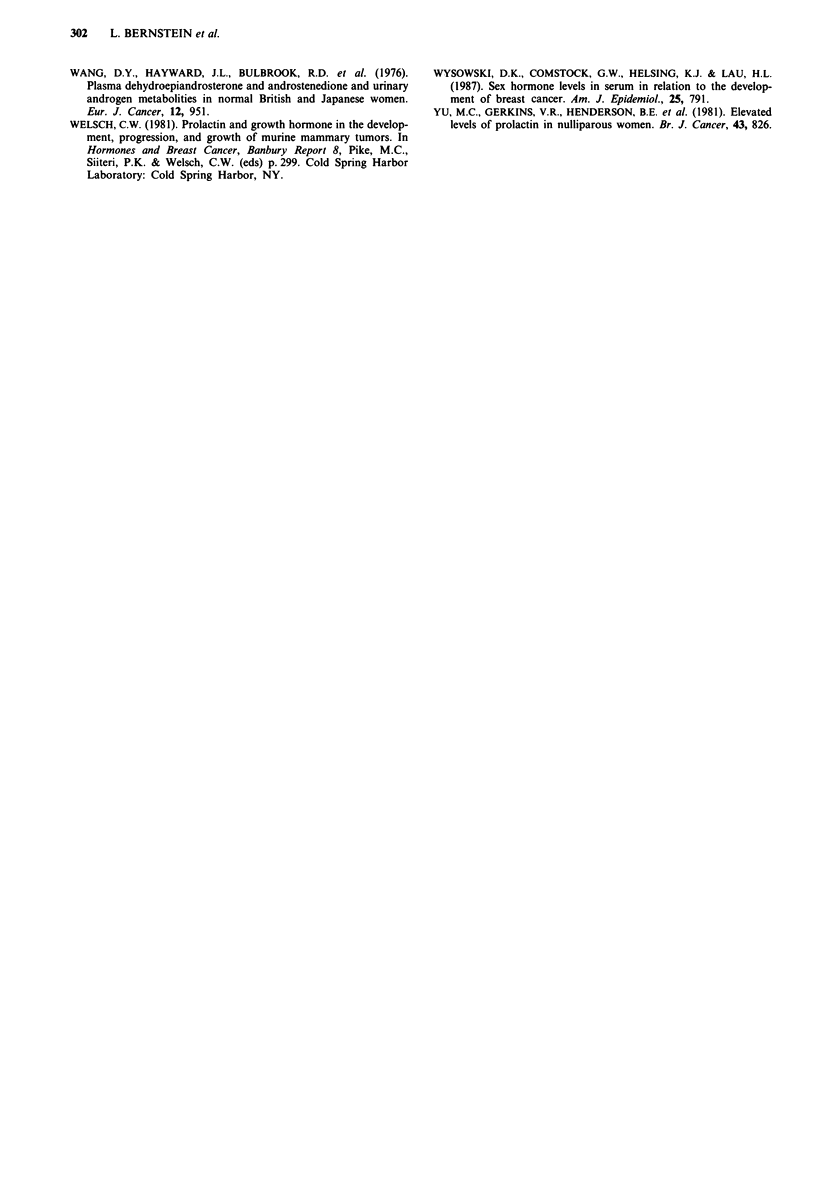# Hormone levels in older women: a study of post-menopausal breast cancer patients and healthy population controls.

**DOI:** 10.1038/bjc.1990.56

**Published:** 1990-02

**Authors:** L. Bernstein, R. K. Ross, M. C. Pike, J. B. Brown, B. E. Henderson

**Affiliations:** Department of Preventive Medicine, University of Southern California School of Medicine, Los Angeles 90033.

## Abstract

Hormone concentrations in blood and total 12 h urine values were compared between 40 post-menopausal women with breast cancer and 40 control women in a study which carefully controlled for the possible confounding effects of age, weight and pregnancy history by individually matching cases and controls on these factors. Breast cancer cases had received only surgical treatment for their localised disease, which was diagnosed from 1 to 9 years before hormonal evaluation. Cases had 15% higher serum oestradiol levels (P = 0.02), 40% more urinary oestradiol (P = 0.03) and 44% more urinary oestriol (P = 0.04) than control women. Cases also had higher levels of serum and urinary oestrone, but these differences were not statistically significant. The percentages of serum oestradiol not bound to albumin or sex-hormone binding globulin did not differ between cases and controls, nor were there statistically significant differences in the serum levels of prolactin, sex-hormone binding globulin or dehydroepiandrosterone sulphate. These results provide further support for the hypothesis that breast cancer risk is determined in part by post-menopausal serum oestrogen concentration.


 © Macmillan Press Ltd., 1990

### Hormone levels in older women: a study of post-menopausal breast cancer patients and healthy population controls

L. Bernstein[1], R.K. Ross[1], M.C. Pike[1], J.B. Brown[2] & B.E. Henderson[1]

[1]*Department of Preventive Medicine, University of Southern California School of Medicine, 1420 San Pablo Street, PMB A-202, Los Angeles, California 90033, USA;* [2]*Department of Obstetrics and Gynaecology, University of Melbourne, Parkeville, Victoria, Australia.*

**Summary** Hormone concentrations in blood and total 12 h urine values were compared between 40 post-menopausal women with breast cancer and 40 control women in a study which carefully controlled for the possible confounding effects of age, weight and pregnancy history by individually matching cases and controls on these factors. Breast cancer cases had received only surgical treatment for their localised disease, which was diagnosed from 1 to 9 years before hormonal evaluation. Cases had 15% higher serum oestradiol levels ($P=0.02$), 40% more urinary oestradiol ($P=0.03$) and 44% more urinary oestriol ($P=0.04$) than control women. Cases also had higher levels of serum and urinary oestrone, but these differences were not statistically significant. The percentages of serum oestradiol not bound to albumin or sex-hormone binding globulin did not differ between cases and controls, nor were there statistically significant differences in the serum levels of prolactin, sex-hormone binding globulin or dehydroepiandrosterone sulphate. These results provide further support for the hypothesis that breast cancer risk is determined in part by post-menopausal serum oestrogen concentration.

There is a substantial body of epidemiological, experimental and clinical evidence for a role of oestrogens in the aetiology of female breast cancer (Henderson *et al.*, 1988). In premenopausal women, the major source of endogenous oestrogens is the ovary. Menopause signals a marked decline in the amount of circulating oestrogens and this decline is at least part of the explanation for the decreased risk of breast cancer associated with early menopause (Trichopoulos *et al.*, 1972). Although breast cancer incidence continues to rise after menopause in US and European women, the rate of increase in breast cancer incidence decreases substantially. In post-menopausal women, the major source of oestrogen is the peripheral conversion of androstenedione in fat tissue (Grodin *et al.*, 1973). This offers the most probable explanation for the association of obesity with increased risk of breast cancer in post-menopausal women (Lubin *et al.*, 1985).

In women, 40-50% of plasma oestradiol ($E_2$) is bound to sex hormone-binding globulin (SHBG) and all but 2-4% of the remainder is bound to albumin (Anderson, 1974). With the development of relevant laboratory technology, attention has focused on evaluating whether the free, non-protein bound fraction of plasma $E_2$ is higher in women with breast cancer than in other women, as free $E_2$ is considered to be freely diffusible into cells and biologically available to receptors in target tissues (Siiteri, 1980); this may also be true of the non-SHGB bound $E_2$ (Pardridge, 1986). This would further explain the effect of body weight on breast cancer risk in older women as increased weight is not only associated with higher post-menopausal oestrogen levels but also with reduced levels of SHBG and, therefore, with greater 'tissue availability' of oestrogens (Siiteri *et al.*, 1981).

A number of studies have compared endogenous oestrogen and other hormone levels in post-menopausal breast cancer cases and in control women by measuring these hormones in blood or urine. Key and Pike (1988) have provided a comprehensive summary of this literature. These studies generally have shown that breast cancer cases have higher levels of oestrogen, lower levels of SHBG, and a larger percentage of non-protein bound $E_2$ than controls. The outcome of statistical tests in these studies has been mixed. This lack of consistent statistical findings appears to be due, in part, to certain aspects of the design of these studies as they have varied in terms of sample size (many have been conducted on small samples), matching criteria (few have matched on age and many do not even report weight), the stage distribution among the cases, the timing of the study in relationship to treatment and the comparability of case and control source populations. With special attention to each of the potential problems of such studies, we have conducted a carefully designed study of blood and urinary hormones in older post-menopausal breast cancer cases and individually matched control women.

Correspondence: L. Bernstein.
Received 23 May 1989; and in revised form 18 August 1989.

### Subjects and methods

### *Subjects*

Breast cancer cases were identified from three health districts in the southern section of Los Angeles County using information collected by the Cancer Surveillance Program (Hisserich *et al.*, 1975), the population-based cancer registry for Los Angeles County, and by the cancer registry of a Southern California retirement community located in adjacent Orange County (Ross *et al.*, 1980). A woman was eligible for the study if her diagnosis of breast cancer had been made at least 1 year after her last menstrual period, if she had localised disease (versus regional disease or distant metastases) that was treated only by surgery, if at least 6 months had passed since surgery for her breast cancer, if she had had no recurrence of her breast cancer and if she had intact ovaries. Case eligibility was further restricted to exclude women with a history of thyroid disease and women who had more than 1 year total lifetime use of exogenous oestrogens or who had used exogenous oestrogens at all in the past 12 months. Forty women with breast cancer were recruited to participate in the study. All women were non-Hispanic white women born in the USA.

Controls were selected from a cohort of women that we are currently following at the retirement community. Restricting selection to women in this community provided a comparable match to the social class of cases as both cases from southern Los Angeles County and members of this adjacent community tend to be of upper-middle socio-economic status. One control was individually matched to each of the 40 cases in terms of age (within 5 years), weight (within 5 kg, although in two cases it was necessary to expand this restriction), and pregnancy history (never pregnant; at least one pregnancy, but no full-term pregnancy; at least one full-term pregnancy). We also matched on height (within 2 cm). Restrictions applied to the cases in terms of thyroid disease and use of exogenous oestrogens were also applied to the controls. As with cases, control women were US born non-Hispanic whites.

### *Methods*

Blood samples were collected from subjects in their homes between 30 and 90 min after the subject awakened in the morning. Each blood drawing consisted of collecting four 5 ml samples which were drawn from an indwelling catheter at 15 min intervals. Samples were collected into sterile tubes without preservative. After centrifugation, the serum was separated and stored in four 2 ml aliquots at −20°C. Before shipping, the samples were thawed and 1 ml from each of the four samples was pooled. Serum samples were shipped on dry ice to Endocrine Sciences Laboratory (Tarzana, CA, USA) for prolactin (unpooled portions of sample 1 and sample 4) and dehydroepiandrosterone sulphate (DHEA-S) (unpooled portion of sample 2) assays and to the laboratory of Howard L. Judd (University of California, Los Angeles, USA) for measurement of oestrone ($E_1$), $E_2$, percentage of free $E_2$ and SHBG using the pooled 4 ml sample. The identities of the specimens were not known to the laboratories; the only identifier was a coded number unique for each specimen; all assays were done at one time.

$E_1$ and $E_2$ were measured by the method of Devane *et al.* (1975). Serum samples were extracted with diethyl ether, chromatographed over microcelite columns and assayed using antiserum developed against oestriol 3, 16, 17-trihemisuccinate. The intra-assay coefficients of variation (CV) based on control pools assayed concurrently were 11.2% for $E_1$ and 17.5% for $E_2$. The percentage of free $E_2$ was determined by the equilibrium dialysis method of Pardridge and Mietus (1979); the intra-assay CV using concurrent control pools was 12.9%. SHBG-binding capacity was measured by the selective ammonium sulphate precipitation technique with the use of a $^3$H-dihydrotestosterone reference (Rosner, 1972). For SHBG-binding capacity, the intra-assay CV for concurrent control pools was 9.5%. Prolactin concentrations were measured by the method of Ehara *et al.* (1973) and DHEA-S was measured directly on diluted serum samples after hydrolysis with sulphatase and using a highly specific antiserum made to DHEA-7-oxine conjugate. The intra-assay CVs were 10.2% for prolactin and 7.5% for DHEA-S.

Overnight urine specimens (12-h) were collected, with collection beginning the evening before the morning that blood was drawn. Urine collection was completed by 40 cases and 37 controls. The urine was treated with 15 ml of 20% acetic acid, divided into 25 ml aliquots and stored at −20°C. Aliquots of urine were coded and air freighted frozen on dry ice to Melbourne, Australia (J.B.B.) where urinary levels of $E_1$, $E_2$ and oestriol ($E_3$) were measured using a method involving spectrophotofluorimetry and internal radioactive standards (Brown, 1976). The intra-assay CVs were 10% for $E_1$, 15% for $E_2$ and 15% for $E_3$. Urine hormone concentrations were converted into absolute amounts by multiplying the concentration by the total volume of urine collected.

The amount of free $E_2$ was computed as the product of total $E_2$ and percentage of free $E_2$. Hormone levels were transformed to logarithmic (base 10) values to achieve approximate normality of distributions for statitical analysis, and geometric mean levels (and 95% confidence limits) are presented in the tables that follow. Quetélet's index was calculated for each woman as the ratio of weight (kg) to the square of height (m$^2$). Paired *t* tests were used to test for differences in geometric mean hormone values between breast cancer cases and matched control women. The relationships of the logarithm of hormone levels to age, age at menopause, weight and time of day that the first blood sample was drawn were assessed by graphical methods and by standard regression techniques and found not to differ significantly from linear. Repeated measures (i.e. matched case–control) analysis of covariance methods were used to test for differences in geometric mean hormone levels adjusted for these factors. The statistical analyses related to urinary hormone levels were restricted to the 37 matched pairs for whom we had adequate urine samples. All *P* values presented are two-sided.

### Results

Characteristics of breast cancer cases and individually matched control women are presented in Table I. On average, breast cancer cases were 1.5 years younger than their respective controls (case age range 53–79; control age range 56–81 years). In all other aspects, the two groups of women were similar. Breast cancer cases had been diagnosed from 1 to 9 years before hormonal evaluation and the mean elapsed time since diagnosis was 4.1 years. Blood drawing was begun between 07.30 and 08.50 for all subjects.

The differences in levels of serum $E_2$ and amounts of urinary $E_2$ and $E_3$ between breast cancer cases and matched controls were statistically significant (Table II). Cases had 14.6% higher levels of serum $E_2$ ($P = 0.02$), 40.0% more urinary $E_2$ ($P = 0.03$) and 43.5% more urinary $E_3$($P = 0.04$) than did control women. Serum and urinary $E_1$ measurements were also greater in cases than controls (10.6% and 23.1% respectively), but these differences were not statistically significant. In terms of total urinary amounts of the three oestrogens assayed, the levels of breast cancer cases exceeded those of controls by 36.9% ($P = 0.04$). No difference between cases and controls was observed in terms of SHBG or in the percentage of serum $E_2$ that was free (i.e. not bound to protein), so that the observed difference in the amount of free $E_2$ (14.1% excess in cases) is solely a function of higher total $E_2$ levels in the cases.

The geometric mean prolactin levels of cases were 10.1% lower than those of controls. This difference was reduced to 1.0% after adjustment was made for the time that blood samples were drawn. Cases also had lower levels of DHEA-S than did control women, but this difference was not statistically significant.

Statistical adjustments for age, age at menopause and weight in the analyses comparing oestrogen and SHBG levels in cases and controls did not alter the results presented in Table II. For urinary oestrogen levels, these adjustments accentuated slightly the differences in means observed in the univariate analyses.

### Discussion

Previous studies have evaluated either urinary oestrogen excretion (Persson & Risholm, 1964; Marmorston *et al.*, 1965; Gronroos & Aho, 1968; Argeulles *et al.*, 1973; Grattarola *et*

**Table I** Mean values (± standard deviation) of relevant characteristics of 40 post-menopausal women with breast cancer and individually matched healthy population controls

| | *Study group* | |
|---|---|---|
| *Characteristic* | *Breast cancer cases* | *Controls* |
| Age at sampling (years) | 68.8 ± 7.6 | 70.3 ± 6.9 |
| Age at last menstrual period (years) | 49.8 ± 5.0 | 49.9 ± 3.8 |
| Years since breast cancer diagnosis | 4.1 ± 2.3 | – |
| Weight (kg) | 63.7 ± 12.0 | 63.2 ± 10.6 |
| Height (m) | 1.64 ± 0.07 | 1.64 ± 0.06 |
| Quetélet's index (kg m$^{-2}$) | 23.7 ± 3.6 | 23.5 ± 3.1 |
| Time samples collected (hours past midnight) | 8.3 ± 0.3 | 8.2 ± 0.3 |

**Table II** Geometric mean values (95% confidence limits) of serum hormones and urinary estrogens of 40 post-menopausal women with breast cancer and individually matched healthy population controls

| | *Study group* | | | |
|---|---|---|---|---|
| *Hormone factor*[a] | *Breast cancer cases* | *Controls* | *Percentage difference*[b] | *Two-sided P value*[c] |
| *Serum* | | | | |
| $E_1$ (pmol $dl^{-1}$) | 8.88 (7.77, 10.13) | 8.03 (6.18, 9.51) | 10.6 | 0.30 |
| $E_2$ (pmol $dl^{-1}$) | 2.57 (2.32, 2.83) | 2.24 (2.03, 2.47) | 14.6 | 0.02 |
| Per cent free[d] | 1.18 (1.11, 1.24) | 1.19 (1.11, 1.27) | −0.8 | 0.77 |
| Amount free (pmol $l^{-1}$) | 2.97 (2.61, 3.41) | 2.61 (2.31, 2.94) | 14.1 | 0.08 |
| SHBG (nmol $l^{-1}$) | 42.5 (37.3, 48.5) | 40.3 (34.9, 46.5) | 5.5 | 0.60 |
| Prolactin[e] (μg $l^{-1}$) | 9.65 (8.15, 11.44) | 10.73 (8.85, 13.00) | −10.1 | 0.43 |
| DHEA-S[e] (nmol $dl^{-1}$) | 116.5 (87.6, 154.6) | 125.7 (95.8, 165.4) | −7.4 | 0.65 |
| *Urine*[f] (nmol 12 $h^{-1}$): | | | | |
| $E_1$ | 59.2 (51.4, 68.4) | 48.1 (37.0, 62.5) | 23.1 | 0.16 |
| $E_2$ | 43.7 (37.4, 50.7) | 31.2 (24.6, 40.4) | 40.0 | 0.03 |
| $E_3$ | 111.0 (92.6, 133.1) | 77.3 (58.9, 101.2) | 43.5 | 0.04 |
| $E_1 + E_2 + E_3$ | 218.6 (189.9, 252.0) | 159.7 (124.6, 204.8) | 36.9 | 0.04 |

[a]$E_1$,oestrone; $E_2$,oestradiol; $E_3$,oestriol; SHBG, sex-hormone binding globulin; DHEA-S, dehydroepiandrosterone sulphate. [b]Percentage difference in geometric means = (case − control)/control. [c]Paired *t* test. [d]Arithmetic mean. [e]Based on 34 matched pairs. [f]Based on 37 matched pairs.

*al.*, 1974; Thijssen *et al.*, 1975; Morreal *et al.*, 1979) or oestrogen concentrations in the blood (England *et al.*, 1974; McFadyen *et al.*, 1976; Malarkey *et al.*, 1977; Adami *et al.*, 1979; Drafta *et al.*, 1980; Moore *et al.*, 1982; Reed *et al.*, 1983, 1985; Secreto *et al.*, 1983; Bruning *et al.*, 1985; Siiteri *et al.*, 1986; Wysowski *et al.*, 1987) of breast cancer cases and controls. These studies have suggested that post-menopausal breast cancer cases have higher endogenous oestrogen levels than controls: five of the seven studies of urinary oestrogen levels found evidence of greater oestrogen excretion in cases (Persson & Risholm, 1964; Marmorston *et al.*, 1965; Arguelles *et al.*, 1973; Grattarola *et al.*, 1974; Morreal *et al.*,1979); of the studies comparing blood levels of $E_1$ and $E_2$ in cases and controls, three of five found higher $E_1$ (Adami *et al.*, 1979; Drafta *et al.*, 1980; Reed *et al.*, 1983) and eight of 11 found higher $E_2$ levels in cases (England *et al.*, 1974; McFadyen *et al.*, 1976; Malarkey *et al.*, 1977; Drafta *et al.*, 1980; Moore *et al.*, 1982; Reed *et al.*, 1985; Bruning *et al.*, 1985; Siiteri *et al.*, 1986). The results of the present study provide evidence that the higher serum oestrogen concentrations of breast cancer cases are mirrored in the urine by higher urinary oestrogen excretion. Unlike most previous studies, cases and controls were closely matched on weight in our study, providing evidence that observed differences in endogenous oestrogen production or metabolism between women with breast cancer and control women is not due entirely to differences in body weight.

Attention has focused recently on measuring the distribution of $E_2$ binding to proteins in blood, following Siiteri's suggestion that the risk of breast cancer may be increased because of tissue exposure to higher levels of circulating, biologically available $E_2$ (Siiteri, 1980). Several recent studies have found that breast cancer cases have higher percentages of free $E_2$ or greater amounts of free $E_2$ than controls (Moore *et al.*, 1982, 1986; Reed *et al.*, 1983, 1985; Bruning *et al.*, 1985; Langley *et al.*, 1985; Ota *et al.*, 1986; Siiteri *et al.*, 1986; Jones *et al.*, 1987). Because higher weight is associated with lower levels of SHBG and higher percentages of both free $E_2$ and non-SHBG bound $E_2$ (Siiteri *et al.*, 1981), the reported higher weight of cases in several of these studies can account, at least in part, for the observed differences. In our study, in which the cases and controls were matched on weight, the average percentage of free $E_2$ of cases and controls did not differ. The fact that cases had about 14% more non-protein bound $E_2$ in their blood than controls simply reflects their greater serum concentrations of $E_2$.

Other hormones have been implicated in breast cancer pathogenesis. In animal studies, prolactin can enhance chemical transformation of breast epithelium and growth of established or transplanted mammary tumours in rodents (Welsch, 1981). After a completed pregnancy, prolactin levels (Yu *et al.*, 1981; Kwa *et al.*, 1981; Musey *et al.*, 1987) as well as oestrogen levels (Bernstein *et al.*, 1985, 1986) are reduced, which may explain the protective effect of a full-term pregnancy on breast cancer risk. Nonetheless, in this study of post-menopausal breast cancer patients, we find no differences in the prolactin levels of cases and controls.

DHEA-S is regarded as an indicator of adrenal androgen secretion (Lobo *et al.*, 1981) and has been shown to be significantly lower in Japanese women (at low risk of breast cancer) than in British women (at high risk of breast cancer) (Wang *et al.*, 1976). It has been proposed that DHEA-S may be involved in the development of breast cancer in older women through the oestrogenic action of a metabolite, 5-androstene-3β,17β-diol, on mammary tissue (Seymour-Munn & Adams, 1983). We observed no significant differences in DHEA-S among breast cancer cases and controls. Cases did, in fact, have lower levels. We evaluated DHEA-S as a measure of adrenal function as it is thought to represent overall function; another measure of adrenal androgen secretion which may provide an explanation for the striking differences in oestrogen levels is androstenedione which is converted to oestrone by muscle and adipose tissue (Tepperman, 1983). Unfortunately, we did not have adequate sera on the majority of subjects to conduct this assay.

This work was supported by grants CA17054 and CA33512 from the National Institutes of Health.

### References

ADAMI, H.O., JOHANSSON, E.D.B., VEGELIUS, J. & VICTOR, A. (1979). Serum concentrations of estrone, androstenedione, testosterone and sex-hormone-binding globulin in postmenopausal women with breast cancer and in age-matched controls. *Upssala J. Med. Sci.*, **84,** 259.

ANDERSON, D.C. (1974). Sex-hormone-binding globulin. *Clin. Endocrinol.*, **3,** 69.

ARGUELLES, A.E., POGGI, U.L., SABORIDA, C. *et al.* (1973). Endocrine profiles and breast cancer. *Lancet*, **i,** 165.

BERNSTEIN, L., DEPUE, R.H., ROSS, R.K. *et al.*, (1986). Higher maternal levels of free estradiol in first compared to second pregnancy: early gestational differences. *J. Natl Cancer Inst.*, **76,** 1035.

BERNSTEIN, L., PIKE, M.C., ROSS, R.K. *et al.* (1985). Estrogen and sex hormone-binding globulin levels in nulliparous and parous women. *J. Natl Cancer Inst.*, **74,** 741.

BROWN, J.B. (1976). Determination of estriol, estrone and estradiol-17$\beta$ in nonpregnancy urine by spectrophotometry or fluorimetry. In *Methods of Hormone Analysis*. Breuer, H., Hamel, D. & Kruskemper, H.L. (eds) p. 446. Wiley: New York.

BRUNING, P.F., BONFRER, J.M.G. & HART, A.A.M. (1985). Non-protein bound oestradiol, sex hormone binding globulin and breast cancer risk. *Br. J. Cancer*, **51,** 479.

DEVANE, G.W., CZELKALA, N.H., JUDD, H.L. *et al.* (1975). Circulating gonadotrophins, estrogens and androgens in polycystic ovarian disease. *Am. J. Obstet. Gynecol.*, **121,** 496.

DRAFTA, D., SCHINDLER, A.F., MILICU, M. *et al.* (1980). Plasma hormones in pre- and postmenopausal breast cancer. *J. Steroid Biochem.*, **43,** 793.

EHARA, Y., SILER, T., VANDEN BERG, G. *et al.* (1973). Circulating prolactin levels during the menstrual cycle: episodic release and diurnal variation. *Am. J. Obstet. Gynecol.*, **117,** 962.

ENGLAND, P.C., SKINNER, L.G., COTTRELL, K.M. & SELLWOOD, R.A. (1974). Serum oestradiol-17$\beta$ in women with benign and malignant breast disease. *Br. J. Cancer*, **30,** 571.

GRATTAROLA, R., SECRETO, G., RECCHIONE, C. & CASTELLINI, W. (1974). Androgens in breast cancer. *Am. J. Obstet. Gynecol.*, **118,** 173.

GRODIN, J.M., SIITERI, P.K. & MACDONALD, P.C. (1973). Source of estrogen production in postmenopausal women. *J. Clin. Endocrinol. Metab.*, **36,** 307.

GRONROOS, M. & AHO, A.J. (1968). Estrogen metabolism in postmenopausal women with primary and recurrent breast cancer. *Eur. J. Cancer*, **4,** 523.

HENDERSON, B.E., ROSS, R.K. & BERNSTEIN, L. (1988). Estrogens as a cause of human cancer: The Richard and Hinda Rosenthal Foundation Award Lecture. *Cancer Res.*, **48,** 246.

HISSERICH, J.C., PRESTON-MARTIN, S. & HENDERSON, B.E. (1975). An areawide cancer reporting network. *Publ. Health Rep.*, **90,** 15.

JONES, L.A., OTA, D.M., JACKSON, G.A. *et al.* (1987). Bioavailability of estradiol as a marker for breast cancer risk assessment. *Cancer Res.*, **47,** 5524.

KEY, T.J.A. & PIKE, M.C. (1988). The role of oestrogens and progestagens in the epidemiology and prevention of breast cancer. *Eur. J. Cancer Clin. Oncol.*, **24,** 29.

KWA, H.G., CLETON, F., BULBROOK, R.D. *et al.* (1981) Plasma prolactin levels and breast cancer: relation to parity, weight and height, and age at first birth. *Int. J. Cancer*, **28,** 31.

LANGLEY, M.S., HAMMOND, G.L., BARDSLEY, A. *et al.* (1985). Serum steroid binding proteins and the bioavailability of estradiol in relation to breast diseases. *J. Natl Cancer Inst.*, **75,** 823.

LOBO, R.A., PAUL. W. & GOEBELSMANN, U. (1981). Dehydroepiandrosterone sulfate (DHEA-S) as an indication of adrenal androgen function. *Obstet. Gynecol.*, **57,** 69.

LUBIN, F., RUDER, A.M., WAX, Y. & MODAN, B. (1985). Overweight and changes in weight throughout adult life in breast cancer etiology. *Am. J. Epidemiol.*, **122,** 579.

MALARKEY, W.B., SCHROEDER, L.L., STEVENS, V.C. *et al.* (1977). Twenty-four-hour preoperative endocrine profiles in women with benign and malignant breast disease. *Cancer Res.*, **37,** 4655.

MARMORSTON, J., CROWLEY, L.G., MYERS, S.M. *et al.* (1965). Urinary excretion of estrone, estradiol, and estriol by patients with breast cancer and benign breast disease. *Am. J. Obstet. Gynecol.*, **4,** 460.

MCFADYEN, I.J., FORREST, A.P.M., PRESCOTT, R.J. *et al.* (1976). Circulating hormone concentrations in women with breast cancer. *Lancet*, **i,** 1100.

MOORE, J.W., CLARK, G.M.G., BULBROOK, R.D. *et al.* (1982). Serum concentrations of total and non-protein-bound oestradiol in patients with breast cancer and in normal controls. *Int. J. Cancer*, **29,** 17.

MOORE, J.W., CLARK, G.M.G., HOARE, S.A. *et al.* (1986). Binding of oestradiol to blood proteins and aetiology of breast cancer. *Int. J. Cancer*, **38,** 625.

MORREAL, C.E., DAO, T.L., NEMOTO, T. & LONEGRAN, P.A. (1979). Urinary excretion of estrone, estradiol and estriol in postmenopausal women with primary breast cancer. *J. Natl Cancer Inst.*, **63,** 1171.

MUSEY, V.C., COLLINS, D.C., MUSEY, P.I. *et al.* (1987). Long-term effect of a first pregnancy on the secretion of prolactin. *N. Engl. J. Med.*, **316,** 229.

OTA, D.M., JONES, L.A., JACKSON, G.L. *et al.*. (1986). Obesity, non-protein-bound estradiol levels, and distribution of estradiol in the sera of breast cancer patients. *Cancer*, **57,** 558.

PARDRIDGE, W.M. (1986). Serum bioavailability of sex steroid hormones. *Clin. Endocrinol. Metab.*, **15,** 259.

PARDRIDGE, W.M. & MIETUS, L.J.(1979). Transport of steroid hormones through the rat brain. *J. Clin. Invest.*, **64,** 145.

PERSSON, B.H. & RISHOLM, L. (1964). Oophorectomy and cortisone treatment as a method of eliminating oestrogen production in patients with breast cancer. *Acta Endocrinol.*, **47,** 15.

REED, M.J., BERANEK, P.A., CHENG, R.W. *et al.* (1985). The distribution of oestradiol in plasma from postmenopausal women with or without breast cancer: relationships with metabolic clearance rates of oestradiol. *Int. J. Cancer*, **35,** 457.

REED, M.J., CHENG, R.W., NOEL, C.T. *et al.* (1983). Plasma levels of estrone, estrone sulfate, and estradiol and the percentage of unbound estradiol in postmenopausal women with and without breast disease. *Cancer Res.*, **43,** 3940.

ROSNER, W. (1972). A simplified method for the quantitative determination of testosterone-estradiol-binding globulin activity in human plasma. *J. Clin. Endocrinol. Metab.*, **34,** 983.

ROSS, R.K., PAGANINI-HILL, A., GERKINS, V.R. *et al.* (1980). A case–control study of menopausal estrogen therapy and breast cancer. *JAMA*, **243,** 1635.

SECRETO, G., RECCHIONE, C., CAVALLERI, A. *et al.* (1983). Circulating levels of testosterone, 17$\beta$-oestradiol, luteinising hormone and prolactin in postmenopausal breast cancer patients. *Br. J. Cancer*, **47,** 269.

SEYMOUR-MUNN, K. & ADAMS, J. (1983). Estrogenic effects of 5-androstene-3$\beta$,17$\beta$-diol at physiologic concentrations and its possible implication in the etiology of breast cancer. *Endocrinology*, **112,** 486.

SIITERI, P.K. (1980). Extraglandular oestrogen formation and serum binding of oestradiol: relationship to cancer. *J. Endocrinol.*, **89,** 119P.

SIITERI, P.K. HAMMOND, G.L. & NISKER, J.A. (1981). Increased availability of serum estrogens in breast cancer: a new hypothesis. In *Hormones and Breast Cancer, Banbury Report 8*, Pike, M.C., Siiteri, P.K. & Welsch, C.W. (eds) p. 87. Cold Spring Harbor Laboratory: Cold Spring Harbor, NY.

SIITERI, P.K., SIMBERG, N. & MURAI, J. (1986). Estrogens and breast cancer. *Ann. NY Acad. Sci.*, **464,** 100.

TEPPERMAN, J. (1983). *Metabolic and Endocrine Physiology*. Year Book Medical Publishers: Chicago.

THIJSSEN, J.H.H., POORTMAN, J. & SCHWARTZ, F. (1975). Androgens in postmenopausal breast cancer: excretion, production and interaction with estrogens. *J. Steroid Biochem.*, **6,** 729.

TRICHOPOULOS, D., MAC MAHON, B. & COLE, P. (1972). The menopause and breast cancer. *J. Natl Cancer Inst.*, **48,** 605.

WANG, D.Y., HAYWARD, J.L., BULBROOK, R.D. *et al.* (1976). Plasma dehydroepiandrosterone and androstenedione and urinary androgen metabolities in normal British and Japanese women. *Eur. J. Cancer*, **12**, 951.

WELSCH, C.W. (1981). Prolactin and growth hormone in the development, progression, and growth of murine mammary tumors. In *Hormones and Breast Cancer, Banbury Report 8*, Pike, M.C., Siiteri, P.K. & Welsch, C.W. (eds) p. 299. Cold Spring Harbor Laboratory: Cold Spring Harbor, NY.

WYSOWSKI, D.K., COMSTOCK, G.W., HELSING, K.J. & LAU, H.L. (1987). Sex hormone levels in serum in relation to the development of breast cancer. *Am. J. Epidemiol.*, **25**, 791.

YU, M.C., GERKINS, V.R., HENDERSON, B.E. *et al.* (1981). Elevated levels of prolactin in nulliparous women. *Br. J. Cancer*, **43**, 826.